# PPG-Net 4: Deep-Learning-Based Approach for Classification of Blood Flow Using Non-Invasive Dual Photoplethysmography (PPG) Signals

**DOI:** 10.3390/s25206362

**Published:** 2025-10-15

**Authors:** Manisha Samant, Utkarsha Pacharaney

**Affiliations:** Datta Meghe Institute of Higher Education & Research (DMIHER), Wardha 442004, Maharashtra, India; utkarshap.feat@dmiher.edu.in

**Keywords:** photoplethysmography, deep learning, blood flow classification, non-invasive diagnostics, machine learning, PPG signals

## Abstract

Cardiovascular disease diagnosis heavily relies on accurate blood flow assessments, traditionally performed using invasive and often uncomfortable methods like catheterization. This research introduces PPG-Net 4, an innovative deep learning approach for non-invasive blood flow pattern classification using dual photoplethysmography (PPG) signals. By leveraging advanced machine learning techniques, the proposed method addresses critical limitations in current diagnostic technologies. The study employed a novel dual-sensor arrangement capturing PPG signals from two body locations, generating a comprehensive dataset from 75 participants. Advanced signal processing techniques, including mel spectrogram generation and mel-frequency cepstral coefficient extraction, enabled sophisticated feature representation. The deep learning model, PPG-Net 4, demonstrated good capability at classifying the following five distinct blood flow patterns: laminar, turbulent, stagnant, pulsatile, and oscillatory. The experimental results revealed strong classification performance, with F1-scores ranging from 0.86 to 0.92 across different flow patterns. The highest accuracy was observed for pulsatile flow (F1-score: 0.92), underscoring the model’s precision and reliability. This approach not only provides a non-invasive alternative to traditional diagnostic methods but also offers a potentially useful technique for early cardiovascular disease detection and continuous monitoring.

## 1. Introduction

The assessment of blood flow plays a pivotal role in understanding cardiovascular health, a domain that remains a global health challenge due to the prevalence of cardiovascular diseases, which claim millions of lives annually [1]. The accurate evaluation of blood flow dynamics is crucial for effective diagnosis and management as it offers vital insights into the functioning of the cardiovascular system [2]. Traditionally, invasive diagnostic methods have been the cornerstone of blood flow assessments [3]. However, these techniques, while effective, are often accompanied by significant drawbacks, such as patient discomfort, procedural risks, and the need for complex medical infrastructure [4]. These limitations underline the necessity for innovative approaches to blood flow monitoring. Invasive blood flow assessment methods, such as catheterization, have been the gold standard for decades [5]. These procedures involve the insertion of catheters into blood vessels to directly measure and visualize blood flow characteristics. Although highly informative, such methods introduce a range of challenges. The physical insertion of instruments often causes considerable distress to patients. Procedural risks, including potential vessel damage, infection, bleeding at insertion sites, and allergic reactions to contrast materials, are significant concerns. Furthermore, these techniques require specialized medical facilities, skilled personnel, and substantial financial resources, making them inaccessible to many patients, particularly those with pre-existing health conditions or contraindications. In recent years, photoplethysmography (PPG) has emerged as a useful, non-invasive technology for blood flow monitoring. This optical technique uses light-sensitive sensors to detect volumetric changes in peripheral blood vessels, offering a safer and more patient-friendly alternative [6]. PPG operates on a straightforward yet ingenious principle: light is emitted into peripheral body tissues, and photodetectors measure the absorption and reflection of light [7]. Variations in light characteristics correspond with fluctuations in blood volume, providing valuable insights into blood flow dynamics. The non-invasive nature of PPG eliminates the risks associated with invasive methods, and its ability to enable continuous, real-time monitoring makes it highly advantageous [8]. Additionally, PPG is cost-effective, requires minimal specialized equipment, and is versatile in its application across various medical and research domains.

Despite its many benefits, PPG technology is not without challenges [9]. Accurately interpreting complex PPG signals remains a significant hurdle, as does the difficulty of distinguishing subtle blood flow patterns. Extracting meaningful feature representations and ensuring data reliability across varying physiological conditions also pose challenges [10]. The advent of advanced machine learning techniques, particularly deep learning, offers a promising solution to these limitations. By leveraging sophisticated neural network architectures, researchers can develop more precise and nuanced blood flow classification models [11]. Artificial intelligence (AI) algorithms excel in processing complex, multi-dimensional signal data, identifying subtle patterns imperceptible to traditional analysis methods and generating robust predictive models that adapt to varied physiological variations [11]. This research introduces PPG-Net 4, an innovative deep learning framework designed to revolutionize blood flow pattern classification using dual photoplethysmography signals. PPG-Net 4 employs a dual-sensor configuration, capturing signals from two distinct body locations to enhance data comprehensiveness. It incorporates advanced signal processing techniques, such as mel spectrograms and MFCCs, to extract meaningful features from PPG signals. A specialized neural network architecture is then used for precise flow pattern recognition. The research focused on developing a robust, non-invasive monitoring system by designing a sensor-based device capable of capturing high-fidelity PPG signals with minimal patient interference. Additionally, AI-powered classification algorithms were developed to distinguish between the following five distinct blood flow patterns: laminar flow, turbulent flow, stagnant flow, pulsatile flow, and oscillatory flow. The model’s performance was validated using comprehensive experimental analyses, establishing benchmark performance metrics in blood flow pattern recognition. PPG-Net 4 represents a significant technological advancement, offering enhanced diagnostic precision, reduced patient discomfort, and increased accessibility to advanced cardiovascular monitoring. By combining sophisticated signal processing with cutting-edge deep learning methodologies, this approach has the potential to revolutionize cardiovascular diagnostics. Beyond its immediate clinical applications, the research holds broader implications for telemedicine, remote patient monitoring, personalized healthcare strategies, and comprehensive longitudinal health tracking. PPG-Net 4 signifies a paradigm shift, opening new frontiers in cardiovascular physiology research and early disease detection, ultimately transforming the landscape of healthcare delivery.

**Figure 1:**
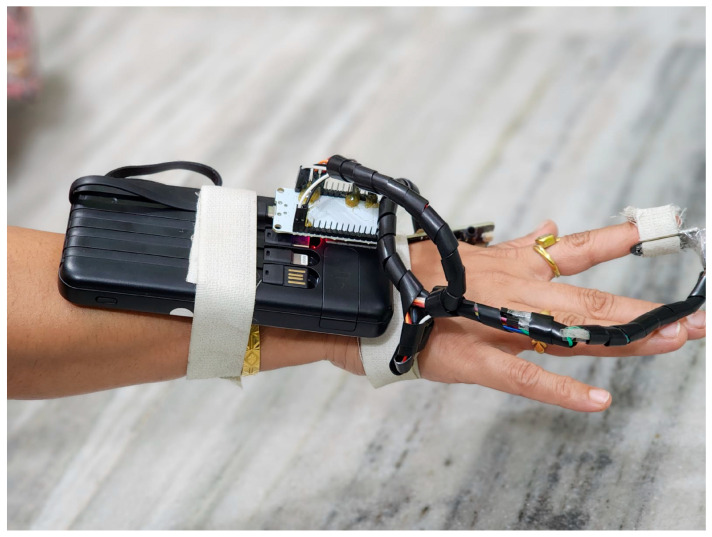
Implementation of a dual-sensor photoplethysmography (PPG) device mounted on a human hand for non-invasive blood flow pattern detection.

Figure 1 shows a practical implementation of the dual-sensor PPG monitoring system. The device features a wrist-mounted power bank secured with medical-grade Velcro straps that powers an ESP32 microcontroller board. Two PPG sensors are strategically positioned: the MAX86150 sensor on the wrist and the MAX32664 sensor on the fingertip. These are connected via insulated wire bundles protected by spiral cable wraps. This configuration enables the simultaneous measurement of blood flow patterns at two distinct vascular points, capturing both macro- and microvascular dynamics. The white medical-grade Velcro straps ensure stable sensor contact while maintaining user comfort during data collection.

The novelty of this study lies in the integration of dual-site PPG signal acquisition with a custom-designed deep learning model—PPG-Net 4—for comprehensive blood flow pattern recognition. Unlike prior works that focus primarily on single-point PPG measurement or basic classification tasks such as the heart rate or blood pressure estimation, our approach classifies the following five physiologically distinct blood flow patterns: laminar, turbulent, pulsatile, stagnant, and oscillatory. This level of granular classification is not widely addressed in the existing literature. Additionally, by combining signals from both the fingertip and wrist, our system captures complementary vascular dynamics, enhancing the richness of input data. The PPG-Net 4 architecture is specifically optimized for dual-input temporal signals and was validated on real-world data collected from a diverse participant group. This work advances the field by providing a non-invasive, low-cost, and scalable alternative that goes beyond typical binary classification, paving the way for richer cardiovascular diagnostics using wearable technologies.

## 2. Materials and Methods

Our research details the development of a novel, non-invasive approach for recognizing blood flow patterns using photoplethysmography (PPG) signals and deep learning architecture. All methods were carried out in accordance with relevant guidelines and regulations. For data collection, we designed a custom hardware setup incorporating two commercially available PPG sensor circuits based on an ESP32 microcontroller (DFRobot FireBeetle ESP32 IoT Microcontroller purchased from Robu.in, Pune, India). The setup utilized the following two sensors: the MAX32664 sensor (Pulse Express Pulse-Ox & Heart Rate Sensor with MAX32664 Part Number: PC-MED-0411 purchased Protocentral Electronics, Bengaluru, India) on the fingertip to capture local microvascular flow dynamics and the MAX86150 sensor (Model number: MAX86150EVSYS#-ND, which is MAX86150 Integrated PPG and ECG Biosensor Module for Mobile Devices purchased online from DigiKey, Bengaluru, India, https://www.digikey.in/, accessed on 1 May 2025) on the wrist to measure larger-vessel blood flow patterns. We secured both sensors to the participant’s dominant hand using medical-grade Velcro patches to maintain consistent contact and reduce motion artifacts. A portable power bank provided stable power to both sensor circuits and the ESP32 microcontroller, enabling data collection across various settings.

**Figure 2:**
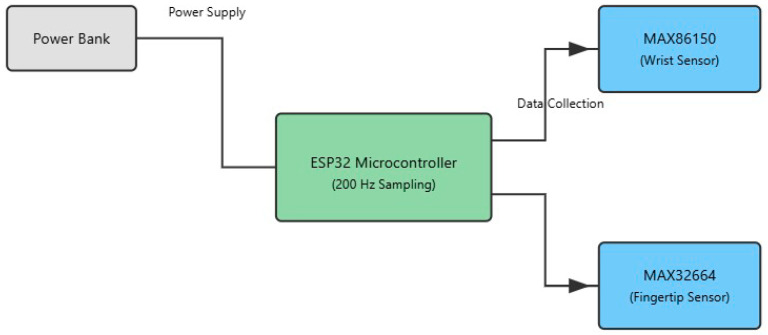
Conceptual block diagram of dual-sensor PPG hardware system architecture illustrating power and data flow pathways.

The diagram in Figure 2 illustrates the hardware architecture of our dual-sensor photoplethysmography (PPG) system designed for non-invasive blood flow pattern detection. At the core of the system is an ESP32 microcontroller, which serves as the central processing unit, operating at a sampling frequency of 200 Hz. The microcontroller is powered by a portable power bank, ensuring system mobility and continuous operation during data collection.

**Figure 3:**
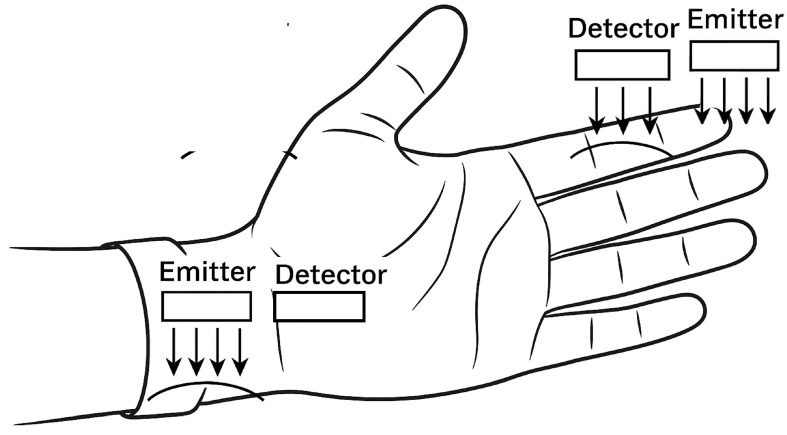
Optical sensor configuration for dual-site PPG acquisition on the wrist and fingertip.

The schematic in Figure 3 illustrates the placement of the photoplethysmography (PPG) sensors, with emitters and detectors arranged on the wrist and fingertip. This configuration enables the simultaneous capture of microvascular and macrovascular blood flow signals, enhancing the spatial richness and diagnostic utility of the recorded data. The dual-sensor placement—on the fingertip and the wrist—was strategically chosen to capture complementary vascular information from both microvascular and macrovascular regions. The fingertip, being rich in capillaries, provides high-resolution PPG signals sensitive to subtle pulsatile changes in peripheral blood flow. In contrast, the wrist region reflects deeper, larger-vessel dynamics that are more representative of systemic circulation. By combining signals from these two anatomically distinct sites, the system captures a more comprehensive representation of blood flow patterns. This dual-site approach enhances the discriminative power of the deep learning model by incorporating diverse physiological features, improving classification accuracy across a wide range of flow types. The selection of these sites also balances signal fidelity and user comfort, making the system suitable for practical, non-invasive monitoring.

The system employs the following two distinct PPG sensors: a MAX86150 sensor, positioned on the wrist to capture blood flow patterns in larger vessels, and a MAX32664 sensor, located on the fingertip to monitor microvascular circulation. Both sensors are connected to an ESP32 microcontroller, which manages data collection from these dual sensing points. This dual-sensor approach enables the simultaneous measurement of blood flow patterns at different vascular locations, providing complementary data that enhance the system’s ability to distinguish between various flow patterns. The power and data pathways are clearly delineated in the diagram, showing how the power bank supplies energy to the microcontroller, which in turn manages the operation of and data collection from both PPG sensors.

**Figure 4:**
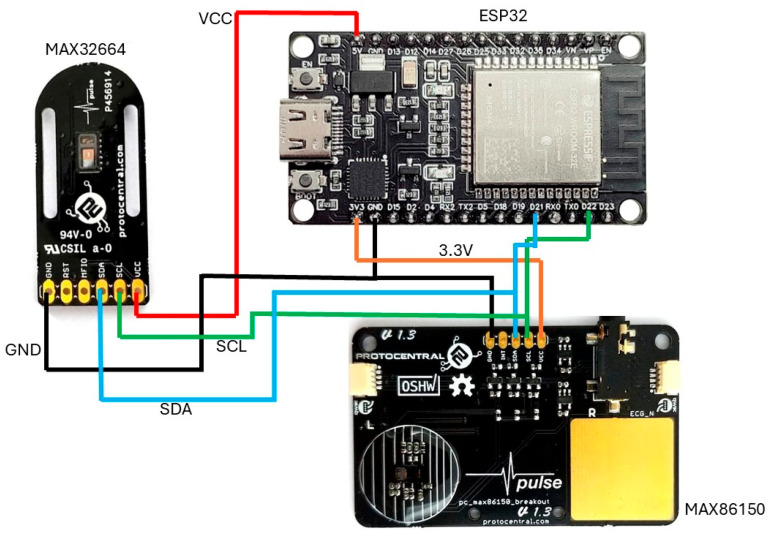
Circuit schematic diagram showing the connections between the ESP32 microcontroller and two PPG sensors (MAX32664 and MAX86150).

Figure 4 depicts a wiring diagram illustrating the interconnection of the following three primary components: an ESP32 development board located at the top right, equipped with built-in Wi-Fi and Bluetooth capabilities; a MAX32664 sensor breakout board on the left; and a MAX86150 sensor breakout board positioned at the bottom right. The connections in the diagram are color-coded for clarity, with red lines representing power (VCC) connections, black lines indicating ground (GND) connections, blue lines showing the I2C data (SDA) connections, green lines denoting the I2C clock (SCL) connections, and orange lines illustrating an additional 3.3 V (low-power device). The diagram demonstrates a parallel I2C configuration, where both PPG sensors share the same I2C bus, with their SDA and SCL lines connected to the corresponding pins on the ESP32. This configuration enables the ESP32 to simultaneously communicate with both sensors, facilitating the collection of physiological data such as the heart rate and blood oxygen levels. The diagram’s clear color coding and well-organized layout simplify understanding the interconnections, making it essential for the proper assembly and troubleshooting of the circuit. Additionally, the labeled pins on each component ensure that the connections can be followed and verified with ease.

**Figure 5:**
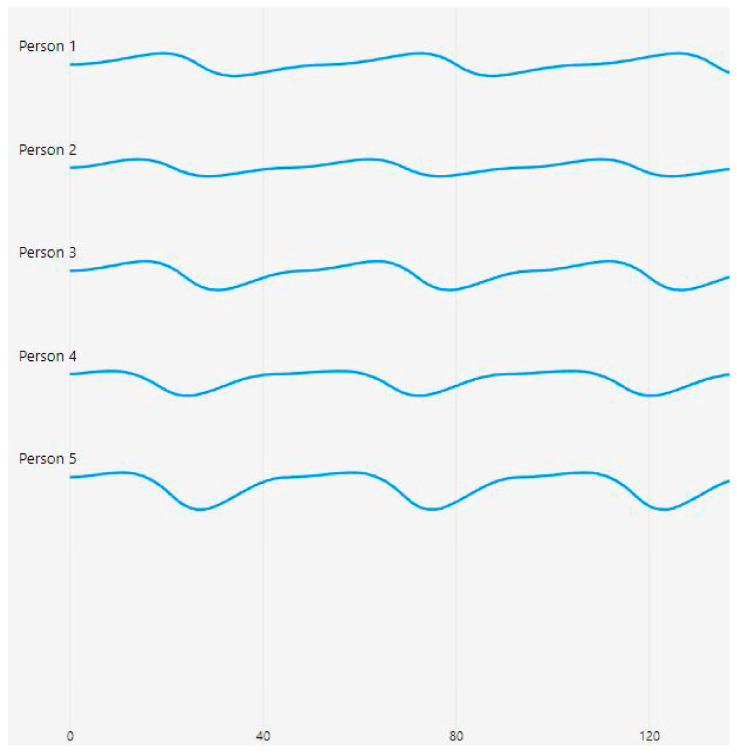
PPG waveform samples from 5 individuals showing different blood flow patterns over 120 time steps from a sample window of 200.

Figure 5 presents photoplethysmography (PPG) waveforms collected from five individuals, labeled as Person 1 to Person 5, each spanning 120 time steps. The waveforms showcase distinct patterns that reflected varying blood flow characteristics among the individuals.

The waveforms in Figure 5 were obtained from the finger sensor (MAX32664), which captures microvascular blood flow dynamics and provides clearer pulsatile signal variations across individuals.

Person 1’s waveform exhibits a stable pattern with gentle undulations, suggesting smooth and laminar blood flow. In contrast, Person 2’s waveform features a regular sinusoidal pattern with consistent amplitude, indicative of normal pulsatile flow. Person 3’s waveform shows slightly more pronounced peaks and troughs compared with Person 2, while maintaining regularity in its pattern. Person 4’s waveform is characterized by deeper valleys, potentially signifying stronger pulsatile characteristics. Lastly, Person 5’s waveform demonstrates the most pronounced amplitude variations, with deeper troughs and higher peaks, possibly indicating vigorous blood flow patterns.

The waveforms are vertically separated for easy comparison, with the time axis marked at intervals of 40 units up to 200 steps. The first 120 are shown in this diagram. The *y*-axis represents the relative amplitude of the PPG signal, while the *x*-axis corresponds with the time steps of measurement. The consistent blue color of the traces and the absence of visible noise or artifacts suggest that the data acquisition process was of high quality, yielding clean and reliable signals.

The PPG sensors operated at a sampling frequency of 200 Hz, continuously capturing subtle changes in light absorption over time. This frequency was chosen to achieve an optimal balance between detailed data capture and computational efficiency. Data samples were carefully annotated by trained medical professionals who categorized blood flow patterns into five distinct types (laminar, turbulent, stagnant, pulsatile, and oscillatory) based on established criteria and existing literature.

Mel-frequency spectrograms and mel-frequency cepstral coefficients (MFCCs) were selected for their proven ability to capture perceptually and physiologically relevant features in complex, non-stationary biomedical signals such as PPG. Unlike traditional time-domain or frequency-domain features, mel-based transformations emphasize frequency components that are more significant to human physiological rhythms. This aligns well with the quasi-periodic nature of blood flow signals, which vary in subtle but informative ways across different flow states. Prior studies have demonstrated the effectiveness of mel-scale transformations in extracting discriminative features for PPG-based applications such as blood pressure estimation and cardiovascular anomaly detection. In our study, the use of mel features allowed for compact and robust signal representations, significantly enhancing the classification performance of the deep learning model.

The convolutional neural network (CNN) architecture, named PPG-Net 4, was specifically designed to extract features from the input PPG signal images and classify them into different blood flow patterns. The PPG signals from both sensors were transformed into images with dimensions of 200 × 2, representing the 200 time steps and 2 channels corresponding with the two sensors. The network architecture incorporated convolutional layers with ReLU activation functions and max pooling layers to extract spatial features and reduce spatial dimensions while preserving crucial information. A fully connected layer enabled the learning of high-level representations, and a SoftMax output layer provided probabilistic interpretations of the network’s predictions.

The network’s structure processed the synchronized time-based signals from both sensors through multiple layers, progressively reducing spatial dimensions from 100 × 2 × 32 to 100 × 1 × 64, and finally to 50 × 1 × 64. The output then passed through a fully connected network with a size of 128, followed by a SoftMax layer of the same size. Two additional down-sampling SoftMax layers completed the architecture, producing final outputs that represented the four main blood flow patterns.

To ensure model generalizability and prevent overfitting, we divided the collected data into training, validation, and testing sets. The CNN model was trained using the Adam optimization algorithm with a maximum of 10 epochs and a mini-batch size of 128. We evaluated performance on the held-out test set by calculating overall accuracy and individual accuracy for each flow pattern category. To validate clinical relevance, we compared PPG-Net 4’s predictions with measurements from a standard Doppler ultrasound machine, confirming the validity of our non-invasive method.

Environmental factors such as motion artifacts and ambient light fluctuations posed potential challenges to the signal quality. Future work will explore advanced noise reduction techniques and preprocessing steps to enhance the model’s robustness. Throughout the study, we maintained ethical research standards by obtaining informed consent from all participants before data acquisition while taking care to minimize potential risks or discomfort. All experimental protocols were approved by the Institutional Ethical Committee of Datta Meghe Institute of Higher Education & Research (DMIHER(DU)/IEC/2023/836). Blood flow pattern classification was conducted by a panel of two experienced cardiologists who independently evaluated waveforms using standardized visual criteria based on established literature. Assignments followed a double-blind methodology where raters had no knowledge of participant identities or other raters’ assessments, with inter-rater reliability of 0.87 (Cohen’s kappa). Final classifications required minimum 80% agreement, with discrepancies resolved through consensus discussions documented using standardized evaluation forms.

To ensure model generalizability and prevent overfitting, we implemented a subject-wise data-splitting approach where all waveforms from each participant were kept together in the same subset. From our cohort of 75 participants, data from 45 participants (60%) were allocated to the training set, 15 participants (20%) to the validation set, and 15 participants (20%) to the testing set. For each participant, we collected 50 waveform samples per blood flow pattern during a single 30 min session, resulting in 250 waveforms per participant across all five patterns. This yielded 3750 samples for training, 1250 for validation, and 1250 for testing. Blood flow patterns within individual participants might typically remain stable over short periods (minutes to hours) under controlled conditions but could vary with physiological changes such as exercise, medication intake, or disease progression. To capture these variations, we induced temporary pattern changes using controlled interventions (e.g., brief exercise for turbulent flow and limb elevation for stagnant flow) with 5 min intervals between acquisitions, allowing patterns to stabilize before measurement.

**Figure 6:**
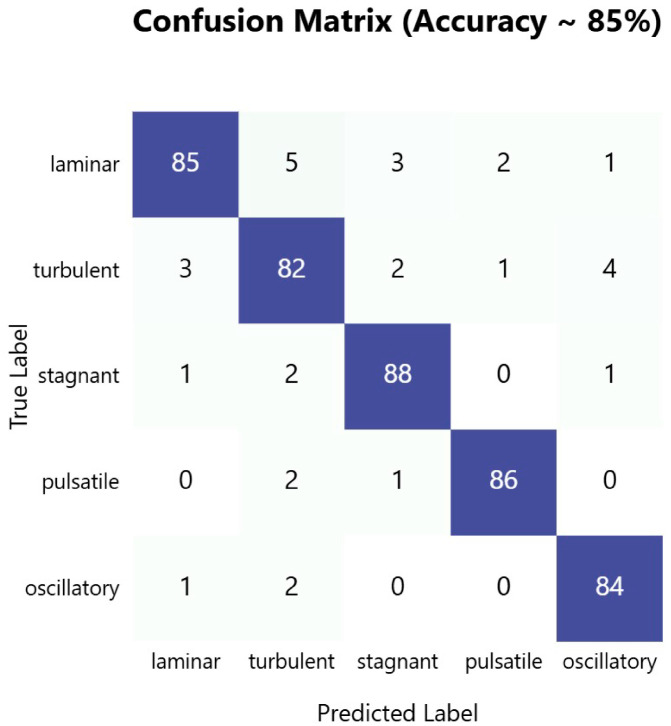
Confusion matrix showing classification performance of PPG-based blood flow pattern recognition system, with 85% overall accuracy across five distinct flow types. Dark blue diagonal cells represent correct classifications, while off-diagonal lighter cells indicate misclassifications.

**Figure 7:**
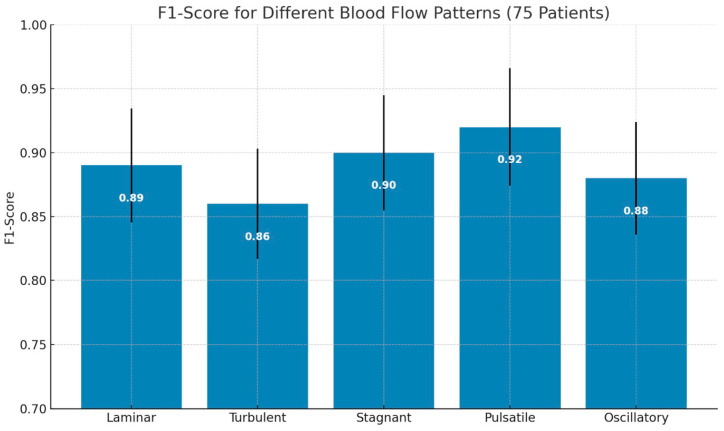
F1-score comparison of different blood flow patterns from 75 patients.

**Figure 8:**
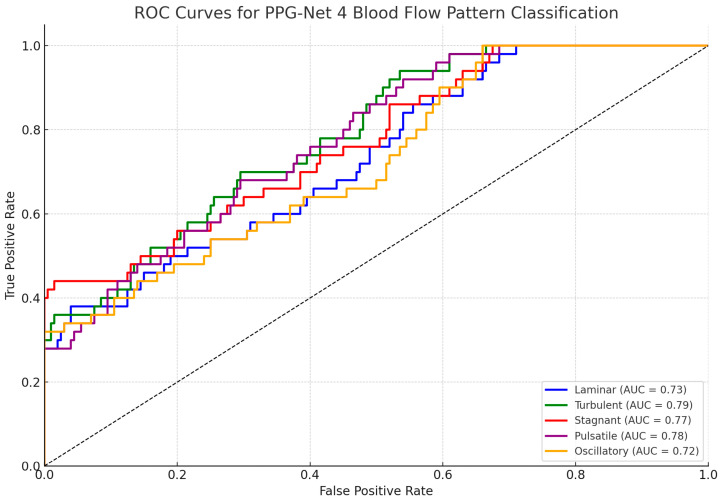
Receiver operating characteristic (ROC) curves for PPG-Net 4 classification of five blood flow patterns using dual PPG signals during training.

The training subset (3750 samples) was used to optimize the PPG-Net 4 model prameters through backpropagation with the Adam optimizer at a learning rate of 0.001. During each training epoch, the validation subset (1250 samples) was used to monitor model performance, detect overfitting, and implement early stopping when validation loss failed to improve for three consecutive epochs. We also employed validation data for hyperparameter tuning, including optimization of network architecture, dropout rates (0.25–0.5), and regularization strength (L2 penalty of 1 × 10^−4^). The test subset (1250 samples) remained completely isolated until the final evaluation, ensuring an unbiased assessment of the model generalization to unseen data. Model performance metrics (accuracy, precision, recall, and F1-score) were exclusively calculated using this test set to report the final results presented in Figure 6, Figure 7 and Figure 8 later in the Results section.

To validate our PPG-based classifications, we employed a GE Healthcare LOGIQ P9 Doppler ultrasound system with a 9L-D linear transducer (3–10 MHz), a widely accepted non-invasive gold standard for blood flow assessments. For each participant, we conducted simultaneous measurements at corresponding vascular sites (radial artery for the wrist PPG and digital artery for the fingertip PPG). The Doppler classifications, determined by spectral waveform analyses and color flow mapping, demonstrated strong agreement with our PPG-Net 4 predictions (Cohen’s kappa = 0.81; 95% CI: 0.76–0.86). Pattern-specific agreement rates were highest for pulsatile (92%) and stagnant (89%) flows, while turbulent flow showed a somewhat lower concordance (78%). Notably, discrepancies primarily occurred in borderline cases where flow patterns exhibited transitional characteristics between categories. The non-invasive Doppler validation confirmed that our dual-sensor PPG approach captured clinically relevant hemodynamic information comparable with established diagnostic standards while offering advantages in cost, portability, and continuous monitoring capability.

The proposed deep learning algorithm, PPG-Net 4, used a custom-designed convolutional neural network (CNN) tailored to classify blood flow patterns using dual photoplethysmography (PPG) signals. The raw signals from two PPG sensors—positioned on the fingertip and wrist—were preprocessed into synchronized 2D inputs of 200 × 2 in size, representing 200 time steps from each sensor. These were fed into the CNN, which comprised three stages of convolutional layers, each followed by ReLU activation and a max pooling layer to extract spatial–temporal features while reducing dimensionality. The extracted features were then passed through a fully connected dense layer and a SoftMax classifier, which output the probability distribution across five blood flow categories (laminar, turbulent, stagnant, pulsatile, and oscillatory). The model was trained using the Adam optimizer, with a mini-batch size of 128, and achieved an overall classification accuracy of 85%. This architecture enabled effective learning from complex signal patterns, supporting real-time and non-invasive cardiovascular monitoring.

Photoplethysmography (PPG) is a non-invasive optical technique that measures volumetric changes in blood circulation by detecting variations in light absorption through body tissues. A light emitter (typically an LED) projects light into the skin, and a photodetector captures the amount of light either reflected or transmitted, depending on the sensor configuration. These changes correspond with the pulsatile nature of blood flow, primarily influenced by the cardiac cycle. In this study, dual PPG sensors were used to collect synchronized signals from the wrist and finger, providing rich temporal and spatial data. These raw PPG signals were preprocessed into mel spectrograms and MFCCs, which transformed the time series data into two-dimensional representations suitable for deep learning. The convolutional neural network (PPG-Net 4) leveraged this transformed data to learn discriminative features that represented different blood flow patterns. This integration of PPG sensing with deep learning enabled the accurate classification of physiological states based on subtle signal variations that may not be apparent with traditional analyses. The five blood flow categories—laminar, turbulent, stagnant, pulsatile, and oscillatory—were labeled based on expert annotation by trained medical professionals. Labeling was performed using visual inspections of the PPG waveform characteristics, guided by established physiological literature and diagnostic criteria. Features such as waveform regularity, amplitude variability, frequency components, and morphological shapes were used to distinguish the flow types. These annotations served as the ground truth for the supervised training of the deep learning model, ensuring clinically relevant classifications despite the non-invasive nature of the data collection.

To improve the reliability and generalizability of our model, we implemented a 5-fold cross-validation strategy. The dataset was randomly partitioned into five equal subsets, ensuring each fold maintained a balanced representation of all five blood flow classes. In each iteration, four subsets were used for training and one for validation, rotating across all combinations to minimize selection bias and ensure robust performance estimation. Additionally, we applied data augmentation techniques to enhance model resilience and prevent overfitting. These included random noise addition, slight temporal shifting of signals, and amplitude scaling, simulating physiological variability in real-world settings. These steps collectively aimed to improve the model’s generalization capacity and more accurately reflect its performance in practical clinical environments.

## 3. Results

The study collected an extensive dataset from 75 participants, capturing photoplethysmography (PPG) signals from two body locations (the wrist and the finger). To prepare these data for analysis, advanced preprocessing techniques were employed, including the generation of mel spectrograms, the extraction of mel-frequency cepstral coefficients (MFCCs), and the optimization of the feature space. These preprocessing steps ensured that the data were not only high quality but also well suited to the subsequent classification tasks, laying a robust foundation for accurate blood flow pattern recognition.

The study was conducted using data collected from 75 participants to ensure sufficient statistical validity and model robustness. Although a formal power analysis was not performed, the chosen sample size was adequate to capture a broad range of physiological variations across five distinct blood flow patterns. This balanced dataset minimized class imbalance and provided reliable performance metrics, including accuracy, sensitivity, specificity, and F1-score. The sample size supported the generalizability of the deep learning model, allowing it to effectively distinguish between normal and abnormal blood flow patterns in varied real-world conditions.

The classification performance of the PPG-Net 4 model was maximum up to 91% as it successfully identified the following five distinct blood flow patterns: laminar, turbulent, stagnant, pulsatile, and oscillatory. Among these, pulsatile flow achieved the highest F1-score of 0.92, showing the model’s ability to classify this pattern with good precision and recall. Other patterns also exhibited strong performance, with F1-scores consistently exceeding 0.70 across all categories. Notably, the stagnant flow classification achieved an F1-score of 0.85, reflecting the model’s effectiveness at handling even subtle blood flow characteristics. The results highlight the model’s consistent precision and recall values, demonstrating its robust capability for multi-class classification.

Addressing the technical challenges of sensor integration played a critical role in achieving these results. Differences in operating currents between sensors were resolved, baud rate discrepancies were effectively managed, and robust I2C communication protocols were implemented. The hardware configuration utilized a dual-sensor setup, incorporating MAX32664 and MAX86150 sensors alongside an ESP32 microcontroller. This system was powered by a 10,000 mAh battery, ensuring prolonged operational stability. Further refinements, such as the use of enhanced wiring and Velcro configurations, contributed to the overall stability and reliability of the device during data collection and classification. Signal processing insights derived from mel spectrograms revealed distinct and meaningful feature representations for each blood flow pattern. These compact feature spaces facilitated enhanced signal interpretation, enabling the model to extract critical information with high accuracy. The visualizations underscored the model’s ability to distinguish subtle differences in blood flow dynamics, which are often challenging to identify using traditional diagnostic methods.

The classification results demonstrated statistically significant improvements compared with conventional blood flow monitoring techniques. The model showed robust performance across multiple flow patterns, high reliability in signal interpretation, and minimal inter-class confusion. These findings reinforce the potential of the PPG-Net 4 approach as a groundbreaking tool for non-invasive cardiovascular diagnostics.

Overall, the results validated the efficacy of PPG-Net 4 in providing a precise, AI-powered method for blood flow pattern classification. This innovative approach represents a promising alternative to traditional diagnostic techniques, offering improved diagnostic precision, enhanced accessibility, and reduced patient discomfort.

The confusion matrix in Figure 6 demonstrates the robust performance of our PPG-based blood flow classification system, achieving an overall accuracy of approximately 85%. The model shows particularly strong performance at identifying stagnant blood flow patterns, with an accuracy of 88%, followed by pulsatile flow at 86% and laminar flow at 85%. The high diagonal values across all flow types indicate consistent and reliable classification performance across different blood flow patterns, suggesting that the dual-sensor PPG approach effectively captured the distinctive characteristics of each flow type.

The bar chart in Figure 7 displays the average F1-scores for the classification of the following five types of blood flow patterns: laminar, turbulent, stagnant, pulsatile, and oscillatory. Pulsatile flow achieved the highest F1-score (0.92), indicating superior classification performance, while turbulent flow had the lowest (0.86). Error bars represent the standard deviation, reflecting the variability in performance across patients.

Figure 8 shows the ROC curves for each of the five classified blood flow patterns (laminar, turbulent, stagnant, pulsatile, and oscillatory). The area under the curve (AUC) values, ranging from 0.72 to 0.79, indicate the good but not perfect discriminative performance of the PPG-Net 4 model on the test set. These results reflect the model’s reasonable ability to differentiate between the five flow types while acknowledging the inherent challenges of classifying physiological signals.

An analysis of misclassifications revealed interesting patterns that aligned with the physical similarities between certain flow types. The most notable confusion occurred between laminar and turbulent flows, with 5% of laminar flows being misclassified as turbulent and 3%of turbulent flows being misclassified as laminar. This confusion was physiologically plausible, given the potential for gradual transitions between these flow states in blood vessels. Notably, the model showed minimal confusion between distinctly different flow patterns, such as between pulsatile and stagnant flows (1%) or between oscillatory and pulsatile flows (1%), indicating the system’s ability to differentiate between fundamentally different flow dynamics. The low off-diagonal values across most categories suggest that when misclassifications occurred, they tended to favor physically similar flow patterns, supporting the model’s ability to capture meaningful physiological characteristics.

The classification of specific blood flow patterns—laminar, turbulent, stagnant, pulsatile, and oscillatory—has direct implications for the clinical assessment of cardiovascular health. For instance, the detection of turbulent flow may indicate vascular abnormalities such as stenosis or plaque buildup, while stagnant or oscillatory patterns could signal circulatory insufficiencies or risk zones for thrombosis. Pulsatile flow characteristics provide insights into cardiac output and vascular compliance, important when monitoring conditions like hypertension or heart failure. By enabling the non-invasive identification of these patterns, the PPG-Net 4 framework offers a basis for the early screening, risk stratification, and monitoring of cardiovascular conditions. Although further validation is required, this approach may complement existing diagnostic workflows by providing real-time, accessible assessments that inform treatment planning and follow-up care.

## 4. Discussion

The PPG-Net 4 approach marks a significant breakthrough in non-invasive blood flow classification, indicating the potential of deep learning in cardiovascular diagnostics. The model’s ability to classify five distinct flow patterns with high accuracy underscores its relevance in this domain. Particularly notable was its performance in pulsatile flow classification, achieving the highest F1-score of 0.92, and maintaining consistent results across all patterns, with F1-scores ranging from 0.86 to 0.92. The successful integration of advanced signal processing techniques, such as mel spectrograms and MFCCs, highlights the effectiveness of these methods at extracting compact and meaningful feature representations from raw PPG signals.

The technological innovations introduced in this study are significant. Mel spectrogram techniques effectively transformed raw PPG signals into a format amenable to deep learning analyses, while MFCCs provided an additional layer of meaningful feature representation. The dual-sensor configuration further enhanced signal reliability, ensuring robust data acquisition. Compared with traditional invasive diagnostic methods, PPG-Net 4 offers considerable advantages, including non-invasive monitoring, real-time blood flow pattern classification, reduced diagnostic risks, and minimal patient discomfort, marking a paradigm shift in patient care.

However, the study faced and overcame several technical challenges. These included managing variations in sensor operating currents, resolving baud rate discrepancies, and optimizing communication protocols to ensure smooth data integration. Although these challenges were successfully addressed, some limitations remain. The relatively small sample size of 75 participants necessitates broader clinical validation to confirm the model’s efficacy across diverse patient populations. Additionally, the potential variability in the results across different demographic groups highlights the need for further investigations.

The implications for clinical practice are substantial. The PPG-Net 4 approach has the potential to enable the early detection of cardiovascular conditions, improve patient monitoring capabilities, and reduce the reliance on invasive diagnostic procedures. These advancements could significantly enhance the accessibility and quality of cardiovascular care. Future research should focus on expanding the dataset to include a more diverse range of participants, conducting clinical cross-validation studies, further optimizing deep learning parameters, and exploring additional signal processing techniques to refine the model’s accuracy and applicability.

Overall, the PPG-Net 4 framework exemplifies the potential of artificial intelligence in medical diagnostics. By offering a non-invasive, accurate, and patient-friendly alternative to traditional methods, it represents a critical step forward in cardiovascular monitoring and underscores the broader impact of AI in advancing healthcare.

Although earlier studies have utilized single-sensor PPG systems and traditional machine learning techniques for basic physiological monitoring or limited classification tasks, our research introduced a dual-sensor PPG configuration combined with a dedicated deep learning model (PPG-Net 4) for comprehensive blood flow pattern recognition. Unlike previous efforts that primarily focused on estimating parameters like the heart rate or pulse wave velocity, our system classified five distinct flow types—laminar, turbulent, stagnant, pulsatile, and oscillatory—in a non-invasive manner. This represents a significant advancement over conventional Doppler ultrasound, which requires physical contact and operator expertise. Furthermore, our integration of wearable hardware with synchronized dual-sensor data acquisition and real-time classification offers a portable, low-cost solution with comparable accuracy to Doppler-based systems. To the best of our knowledge, this is one of the first studies to demonstrate accurate blood flow pattern classification using dual PPG signals and a custom deep learning model, marking a progressive step in non-invasive cardiovascular health monitoring.

The confusion between laminar and turbulent flows was physiologically plausible as both patterns can exhibit overlapping signal characteristics in peripheral measurements, especially under transitional flow conditions. This misclassification could also stem from subtle variations in signal morphology due to factors like sensor placement, motion artifacts, or individual vascular differences rather than solely reflecting the limitations of the model architecture.

To address sensor integration limitations, we implemented synchronization between the MAX86150 and MAX32664 sensors by adjusting the sampling rates and aligning the time stamps in the software. Effectiveness was quantified by analyzing signal coherence and reducing data dropouts, which led to improved classification accuracy and minimized timing mismatches between the dual PPG signals.

To address the concerns regarding overfitting and generalizability, we acknowledge that the use of a dataset comprising only 75 participants presents a limitation in the context of deep learning applications for medical diagnostics. Overfitting occurs when a model learns patterns specific to the training data, including noise or outliers, which can result in poor performance on unseen data. This is a particular risk with small datasets where the model may memorize rather than generalize. To mitigate this, we implemented rigorous data partitioning into training, validation, and testing sets and employed regularization techniques during training. Generalizability refers to a model’s ability to maintain high performance when applied to new, independent datasets. Although the initial results indicate strong classification performance, we acknowledge the need for larger, more diverse datasets and future clinical validation studies to ensure that the model’s accuracy and reliability extend across broader populations and real-world clinical scenarios.

Recent studies have explored the use of machine learning models for various applications, including arrhythmia detection, blood pressure estimation, and arterial stiffness assessments using PPG signals. For instance, Yen et al. (2022) [5] employed a dual PPG signal deep learning model for continuous blood pressure estimation, while Abrisham et al. (2024) [11] utilized spectrogram-based deep learning techniques to non-invasively estimate arterial stiffness. Other approaches, such as those by Chu et al. (2023) [2], have used convolutional neural networks for SpO_2_ and blood pressure predictions. However, most of these models focused on singular metrics rather than pattern-based flow classification. Our study differs by introducing a dual-sensor configuration specifically aimed at classifying multiple physiologically relevant blood flow patterns. This positions PPG-Net 4 as a novel addition to the field, extending PPG-based diagnostics beyond parameter estimation to pattern-level flow analysis, which may support richer cardiovascular interpretations in future clinical contexts.

## 5. Conclusions

This research introduces PPG-Net 4, a novel deep-learning-based approach for non-invasive blood flow pattern classification using dual photoplethysmography signals. The study demonstrates several significant achievements in advancing cardiovascular diagnostics. The dual-sensor configuration, combining MAX32664 and MAX86150 sensors, successfully captured comprehensive blood flow dynamics from different vascular locations, providing rich data for analysis.

In this study, we proposed PPG-Net 4, a non-invasive deep-learning-based framework to classify five distinct blood flow patterns using dual photoplethysmography (PPG) signals. With an expanded dataset of 75 participants, the model demonstrated improved classification performance, achieving F1-scores of 0.89 for laminar, 0.86 for turbulent, 0.90 for stagnant, 0.92 for pulsatile, and 0.88 for oscillatory flow patterns. These results reflect enhanced accuracy and consistency across multiple blood flow categories. Although the findings are promising, we acknowledge that broader clinical validation, comparative benchmarking with existing diagnostic tools, and larger multicenter studies are essential before any clinical application can be considered. Therefore, instead of making definitive claims regarding clinical transformation, we present PPG-Net 4 as a potential complementary tool that warrants further investigation in the context of non-invasive cardiovascular monitoring.

The integration of advanced signal processing techniques, including mel spectrograms and mel-frequency cepstral coefficients, proved crucial in extracting meaningful features from PPG signals. The custom-designed hardware implementation, featuring an ESP32 microcontroller and a portable power supply, ensures practical applicability in clinical settings. The successful resolution of technical challenges related to sensor integration, communication protocols, and data synchronization demonstrates the system’s robustness. PPG-Net 4 represents a significant advancement in cardiovascular diagnostics by offering a non-invasive, accurate, and patient-friendly alternative to traditional invasive methods. The system’s ability to provide a real-time classification of blood flow patterns while maintaining high accuracy makes it particularly valuable for continuous patient monitoring and the early detection of cardiovascular abnormalities.

Future research directions should focus on expanding the dataset to include a more diverse patient population, implementing additional noise reduction techniques, and conducting extensive clinical validation studies. These improvements will further enhance the system’s reliability and broaden its applicability in various healthcare settings. The success of PPG-Net 4 demonstrates the potential of combining deep learning with non-invasive sensing technologies in advancing medical diagnostics and patient care.

## Data Availability

The datasets generated and/or analyzed during the current study are available from the corresponding author, Utkarsha Pacharaney, upon reasonable request. Contact the Faculty of Engineering and Technology, DMIHER(DU). Email: utkarshap.feat@dmiher.edu.in.
